# P-39. How Many Are Missed? Methicillin-Resistant Staphylococcus Aureus Deaths Attributable to Injection Drug Use in the U.S

**DOI:** 10.1093/ofid/ofaf695.268

**Published:** 2026-01-11

**Authors:** Leslie Bull, Kevin Ikuta

**Affiliations:** UCLA, Los Angeles, California; West Los Angeles VA, Los Angeles, California

## Abstract

**Background:**

Injection drug use (IVDU) is associated with potentially fatal health risks, including overdose and infection. However, estimates on the fatal burden attributable to IVDU are limited to deaths due to overdose. By neglecting the infectious sequelae of IVDU, estimates undercount the true burden of IVDU and the urgency of the epidemic. This lack of data makes it difficult to target programs aimed at reducing deaths related to systemic MRSA and other infections among people who inject drugs (PWID). To fill this epidemiologic knowledge gap, we developed a novel method to calculate the burden of MRSA deaths in the U.S. that can be directly attributed to IVDU based on existing, previously published data.

**Figure 1.**
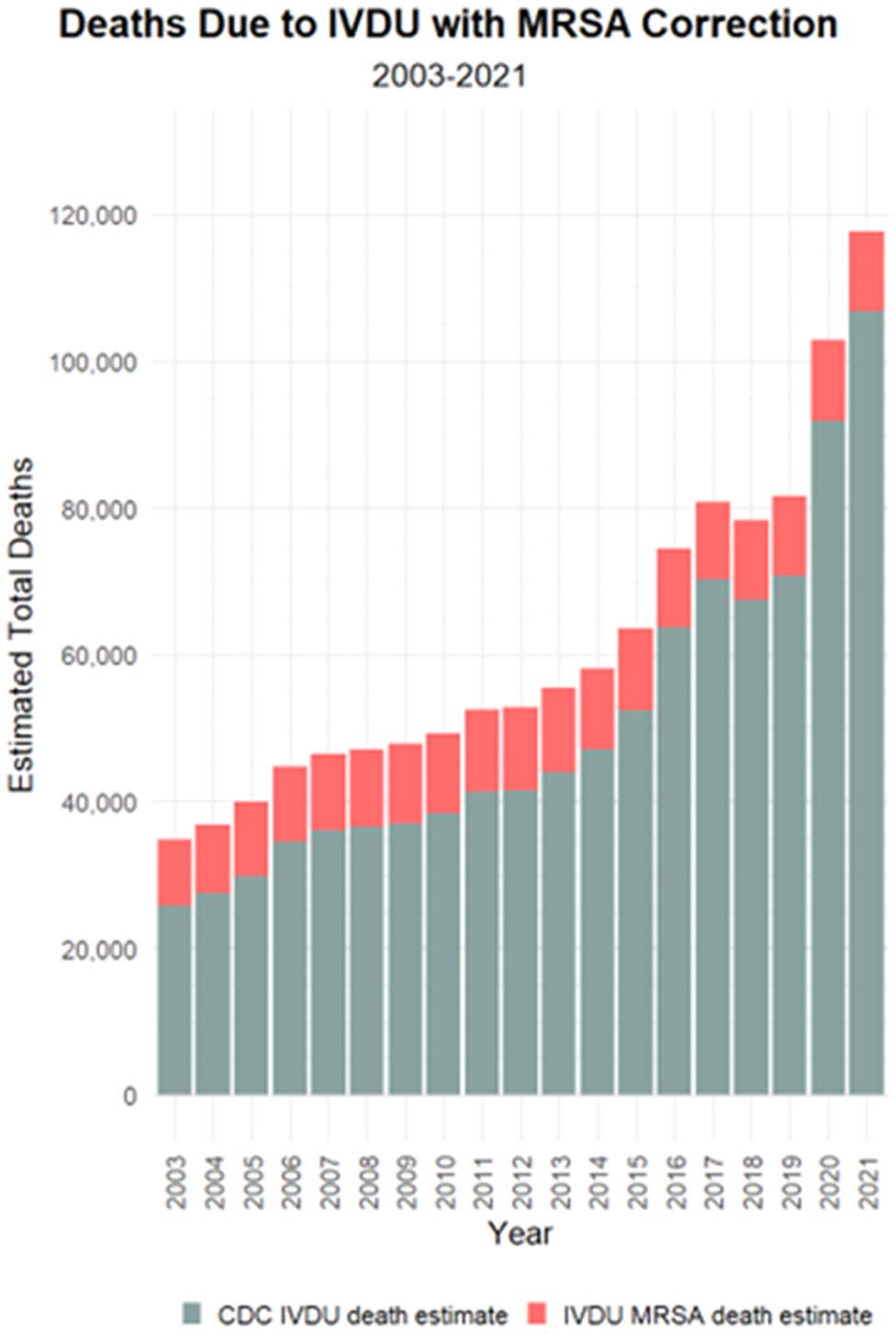
Deaths due to IVDU with MRSA correction Deaths due to IVDU with MRSA Correction

**Methods:**

We estimated MRSA deaths attributable to IVDU using a population attributable fraction (PAF) and MRSA mortality estimates from 1990-2021 extracted from Global Burden of Disease (GBD) estimates. The PAF components were extracted from recent literature on the relative risk (RR) of MRSA in PWID and prevalence of IVDU among MRSA cases(P_e_).

**Results:**

Recent studies found the RR of invasive MRSA infection in PWID was 16 and the P_e_ was 24%. Thus, PAF = (P_e_) * (1-[1/(RR)]) = (0.24) * (1-[1/(16)]) = 0.225. In 2021, there were an estimated 48,980 deaths associated with MRSA. Give this, we estimated 11,021 MRSA deaths directly attributable to IVDU in the US in 2021(22.5% of all MRSA deaths in the US). In total, from 2003 to 2021, we estimate 202,779 total MRSA deaths attributable to IVDU that were not counted in CDC IVDU death totals.

**Conclusion:**

Conclusions: Current health metrics underestimate the fatal burden due to IVDU. Our estimates show that thousands of deaths due to MRSA can be directly attributed to IVDU. While our analysis focused on MRSA due to the ready availability of estimates on prevalence of relative risk in the published literature, there are many other potentially deadly infections associated with IVDU, e.g., HIV and Hepatitis C. Further research is needed to expand this analysis to other infections and pathogens attributable to IVDU to more fully estimate its true health burden.

**Disclosures:**

All Authors: No reported disclosures

